# Prevalence of ESBL-producing Enterobacteriaceae in raw vegetables

**DOI:** 10.1007/s10096-014-2142-7

**Published:** 2014-05-22

**Authors:** E. A. Reuland, N. al Naiemi, S. A. Raadsen, P. H. M. Savelkoul, J. A. J. W. Kluytmans, C. M. J. E. Vandenbroucke-Grauls

**Affiliations:** 1 Department of Medical Microbiology and Infection Control, VU University Medical Center, P.O. Box 7057, 1007 MB Amsterdam, The Netherlands; 2 Laboratory for Medical Microbiology and Public Health, Enschede, The Netherlands; 3 Medical Microbiology and Infection Control, Ziekenhuisgroep Twente, Almelo, The Netherlands; 4 Department of Medical Microbiology and Infection Control, Amphia Hospital, Breda, The Netherlands

## Abstract

To determine whether extended-spectrum beta-lactamase (ESBL)-producing Enterobacteriaceae (ESBL-E) are present in retail raw vegetables in Amsterdam, the Netherlands, we collected 119 samples of 15 different types of vegetables from various sources. After culture, strain identification and susceptibility testing, ESBL-encoding genes were characterised by a microarray. Four of the 15 vegetable types were contaminated with ESBL-E. Seven samples (6 %) yielded ESBL-E. Three *bla*_CTX-M-15_, one *bla*_CTX-M-1_, two genes of the CTX-M-9 group and one SHV ESBL-encoding gene were found. The ESBL genes were similar to what is found in enterobacterial strains from human origin. Therefore, raw vegetables might be a source of resistance genes for the enterobacterial strains found in humans.

## Introduction

Extended-spectrum beta-lactamases (ESBLs) are emerging rapidly worldwide. ESBLs have been detected in patients without prior healthcare contact, even in countries with low consumption of antibiotics (ESAC-Net; http://www.ecdc.europa.eu/en/activities/surveillance/esac-net/pages/index.aspx). Major sources of ESBLs in the community can be envisaged: resistant strains from high ESBL prevalence reservoirs (hospitals and long-term care facilities) or resistant strains present in the food chain, environment or water sources [1]. The complex dynamics and dissemination of antibiotic resistance and its relation to different reservoirs has been depicted by Davies and Davies [2] and by Wellington et al. [3]. Especially, the food chain has recently attracted attention because a high prevalence of resistance genes in food-producing animals like poultry was reported; this is related to the high rate of antimicrobial drug use in the livestock sector [4, 5]. In addition, resistance genes are also widespread in agriculture [2, 6]. Ruimy et al. observed that vegetables in France were often contaminated with resistance genes [7]. In 2011, a Shiga toxin-producing *Escherichia coli* (STEC) outbreak in Germany, caused by ESBL-producing *E. coli*, was traced to sprouts [8].

The aim of this study was to evaluate the presence of ESBL-producing Enterobacteriaceae (ESBL-E) in raw vegetables in the region of Amsterdam, the Netherlands.

## Materials and methods

### Study design

In October, 2010, and March, 2011, 119 samples from 15 different types of retail vegetables were purchased from an organic store, the market, a store of a large Dutch supermarket chain and a local supermarket. We obtained two samples from each vegetable type and from each different source. We focused on vegetables grown on and in the ground/soil, and on (mung) bean sprouts. The 15 vegetables included: beet root, Brussels sprouts, carrots, cauliflower, celery, chicory, cucumber, lettuce, mushrooms, parsnip, potatoes, radish, spinach, spring onion and (mung) bean sprouts. Of each unwashed sample, 1 g was ground and inoculated in 10 ml of trypticase soy broth (Becton Dickinson, Breda, the Netherlands) supplemented with ceftazidime 0.5 mg/L (incubation overnight, at 37 °C). Thereafter, the broth was subcultured on a selective screening agar (EbSA, Cepheid Benelux, Apeldoorn, the Netherlands) (incubation overnight, 37 °C) [9]. We only analysed enterobacterial species.

### Phenotypic and genotypic testing

Identification and antibiotic susceptibility testing were performed with the Vitek 2 system (bioMérieux, Marcy l’Etoile, France). The minimum inhibitory concentration (MIC) breakpoints according to the Clinical and Laboratory Standards Institute (CLSI) were used [10]. Phenotypic ESBL production was confirmed with the combination disc diffusion test with clavulanic acid (Rosco, Taastrup, Denmark) [11].

After DNA isolation (QIAamp DNA mini kit, Qiagen, Venlo, the Netherlands), isolates were screened for ESBL resistance genes by a microarray, which enables the distinction between the most prevalent ESBLs, including CTX-M-1 and CTX-M-15 (Check-MDR CT103, Check-Points, Wageningen, the Netherlands) [12]. The presence of carbapenemase genes and plasmid-mediated AmpCs was also tested with the microarray. Plasmids were identified by polymerase chain reaction (PCR)-based replicon typing [13]. The strains were tested with 22 primer sets for Inc groups (ColE, FIA, ColEtp, HI1, HI2, T, Inc I1, FrepB, R, FIIs, FIB, P, B/O, A/C, K, U, L/M, W, N, X, FIC, Y).

## Results

Seven of the 119 samples (6 %) yielded ESBL-E (Table 1). The type of contaminated vegetables, ESBL-producing strains, genes and plasmids found are described in Table 1. The contaminated samples were from four of the 15 vegetable types (27 %). The majority of ESBL-positive samples (5/7) were derived from an organic store, the prevalence of ESBL-E in organically grown vegetables was 15.6 % (5/32) [95 % confidence interval (CI): 6.4–32.2] and in conventionally grown vegetables it was 2.3 % (2/87) (95 % CI: 0.14–8.5). The risk difference (RD) between the two types of vegetables was significant (RD 13.3 %, 95 % CI: 0.35–26.31). The variation in plasmids and genes found is in accordance with the findings of Carattoli et al. [13].

**Table 1:** Overview of vegetable types in relation to extended-spectrum beta-lactamase (ESBL)-encoding genes and plasmids

Store	Vegetable	Species	ESBL gene	Plasmids
Organic store	Bean sprouts	*Klebsiella pneumoniae*	*bla*_SHV-12_	ColEtp, T
	Bean sprouts	*Klebsiella pneumoniae*	*bla*_CTX-M-14_	ColE, FIA
	Radish	*Enterobacter cloacae*	*bla*_CTX-M-15_	ColEtp, HI2
	Spring onion	*Enterobacter amnigenus*	*bla*_CTX-M-15_	HI2
	Parsnip	*Citrobacter braakii*	*bla*_CTX-M-1_	HI1
Market	Bean sprouts	*Klebsiella pneumoniae*	*bla*_CTX-M-14_	ColEtp, FIA, T
Supermarket (local)	Bean sprouts	*Enterobacter cloacae*	*bla*_CTX-M-15_	ColEtp, HI2

A high rate of co-resistance to gentamicin, co-trimoxazole and nitrofurantoin was observed. Two out of these seven ESBL-producing strains were multi-resistant, but all were susceptible to meropenem and imipenem [14].

## Discussion

Our results document the presence of ESBL-E in retail raw vegetables obtained in Amsterdam, the Netherlands, which implies that raw vegetables may be a source of resistance genes. These results correlate with other studies pointing to vegetables as a possible route for the dissemination of resistance genes in the community [4, 7, 15, 16].

Different pathways might be relevant to explain why these resistance genes were found on raw vegetables [2, 6]. Knapp et al. showed that the levels of antibiotic resistance genes in soil have increased considerably over the past 70 years in the Netherlands [17]. Other reservoirs of antibiotic resistance genes are the aquatic system and sewage, created by antibiotic use and waste disposal [2, 4, 18, 19]. Fresh produce can also become contaminated during processing [15]. We bought the vegetables at the market or at the shop itself, thereby providing information on the vegetables actually bought by the consumer, but this implies that contamination could have occurred during human handling.

A remarkable finding of the present study is that organic vegetables were more often contaminated with ESBL-E than conventionally produced vegetables. Several studies examined the potential differences between organic and conventional products [18]. It has been shown that different microorganisms and contaminants are found on organic produce compared to conventionally grown vegetables due to the use of manure, no pesticides and a different processing [18].

The resistance genes present in our samples belonged mainly to the CTX-M family, and especially CTX-M-15 was found. CTX-M-15 is the most common type of ESBL in Europe and has been increasingly described in community isolates. Also, a study in Amsterdam, among Dutch outpatients with gastrointestinal complaints, showed that the most prevalent ESBL-E is CTX-M-15-producing *E. coli* [20]. A study performed in the region of Rotterdam describes the clinical and molecular characteristics of bacteraemia caused by ESBLs, also showing that these strains are the most prevalent [21]. Therefore, although the species containing these genes were not *E. coli* but other enterobacterial species, these may act as a reservoir for mobile drug resistance genes and transfer these genes to the non-pathogenic *E. coli* present in the gut [22, 23]. The high rate of co-resistance that we noted may add to the acquisition of multi-drug resistance from the food chain.

Sprouts were found to be most often contaminated, but with other ESBL-producing species than *E. coli*. These vegetables have often been implicated in STEC outbreaks and it has been postulated that they may constitute a common gene pool for antibiotic resistance [8].

In a previous study, we found that ESBL-E carried several different plasmids, some of which we also detected in the enterobacterial strains found on vegetables in the present study [20]. The most common plasmids in humans were ColE and FrepB, but ColEtp and HI2 were also found in a substantial number of strains. Hence, plasmid transfer among Enterobacteriaceae found on vegetables and those of the human gut seems possible. It is, of course, not possible, from the present study, to infer how frequent such transfer could be.

In conclusion, we found classic ESBL genes in raw vegetables, namely CTX-M-1 and CTX-M-15, and several plasmids that are commonly associated with these genes. The possible impact of our findings on human health highlights the need to further evaluate the presence of ESBL-E in raw vegetables and to explore whether the exchange of resistance genes between these ESBL-E and other enterobacterial species in the human gut does indeed occur.
